# Protective Efficacy of a Human Endogenous Retrovirus Envelope-Coated, Nonreplicable, Baculovirus-Based Hemagglutin Vaccine against Pandemic Influenza H1N1 2009 

**DOI:** 10.1371/journal.pone.0080762

**Published:** 2013-11-18

**Authors:** Jae-Yoo Choi, Yong-Dae Gwon, Jeong-Ki Kim, Yeon-Dong Cho, Yoon-Ki Heo, Han-Sam Cho, Tae-Jin Choi, Ha-Ryoung Poo, Yu-Kyoung Oh, Young Bong Kim

**Affiliations:** 1 Department of Animal Biotechnology, Konkuk University, Seoul, Republic of Korea; 2 Systemic Proteomics Research Center, KRIBB, Taejeon, Republic of Korea; 3 Department of Microbiology, Pukyong National University, Busan, Republic of Korea; 4 College of Pharmacy, Seoul National University, Seoul, Republic of Korea; Public Health Agency of Canada, Canada

## Abstract

Despite the advantages of DNA vaccines, overcoming their lower efficacy relative to that of conventional vaccines remains a challenge. Here, we constructed a human endogenous retrovirus (HERV) envelope-coated, nonreplicable, baculovirus-based HA vaccine against swine influenza A/California/04/2009(H1N1) hemagglutin (HA) (AcHERV-sH1N1-HA) as an alternative to conventional vaccines and evaluated its efficacy in two strains of mice, BALB/c and C57BL/6. A commercially available, killed virus vaccine was used as a positive control. Mice were intramuscularly administered AcHERV-sH1N1-HA or the commercial vaccine and subsequently given two booster injections. Compared with the commercial vaccine, AcHERV-sH1N1-HA induced significantly higher levels of cellular immune responses in both BALB/c and C57BL/6 mice. Unlike cellular immune responses, humoral immune responses depended on the strain of mice. Following immunization with AcHERV-sH1N1-HA, C57BL/6 mice showed HA-specific IgG titers 10- to 100-fold lower than those of BALB/c mice. In line with the different levels of humoral immune responses, the survival of immunized mice after intranasal challenge with sH1N1 virus (A/California/04/2009) depended on the strain. After challenge with 10-times the median lethal dose (MLD_50_) of sH1N1 virus, 100% of BALB/c mice immunized with the commercial vaccine or AcHERV-sH1N1-HA survived. In contrast, C57BL/6 mice immunized with AcHERV-sH1N1-HA or the commercial vaccine showed 60% and 70% survival respectively, after challenge with sH1N1 virus. In all mice, virus titers and results of histological analyses of lung tissues were consistent with the survival data. Our results indicate the importance of humoral immune response as a major defense system against influenza viral infection. Moreover, the complete survival of BALB/c mice immunized with AcHERV-sH1N1-HA after challenge with sH1N1 virus suggests the potential of baculoviral vector-based vaccines to achieve an efficacy comparable to that of killed virus vaccines.

## Introduction

Influenza A viruses of the *Orthomyxoviridae* family, which have a genome composed of eight segments of negative single-strand RNA, are the causative agents of influenza [1-3]. After infection, two glycoproteins of the influenza virus membrane—hemagglutin (HA) and neuraminidase (NA)—emerge in the body during replication cycles in response to host immunological pressure, resulting in new epidemic strains every 1 to 2 years [4]. There are currently 17 known HA subtypes (H1–17) and nine known NA subtypes (N1–9) with a number of possible combinations between HA and NA (N1-N9) [5,6].

In April 2009, an outbreak of influenza in North America was found to be caused by the swine-origin influenza virus A/California/04/2009(H1N1). The virus has been rapidly spread by people and by August 1, 2010, more than 214 countries and overseas territories worldwide had reported laboratory-confirmed cases of pandemic influenza H1N1 2009, including over 18,449 deaths [7]. Currently, H1N1 influenza has moved into the post-pandemic period and circulates as a seasonal virus. 

Vaccines are considered to be one of the most effective tools for preventing and controlling the spread of pandemic influenza. However, the production of a conventional vaccine against a rapidly emerging new strain takes 3 to 6 months. Moreover, all influenza vaccines currently licensed are produced using embryonated chicken eggs. The use of eggs for the production of influenza vaccine raises several potential problems, including vulnerability of the egg supply, necessity for producing strains selected for high yield growth in eggs, and a contra-indication in people with egg allergies [8]. Several strategies have been explored for overcoming these obstacles. Some efforts have been made to grow influenza viruses in cell lines [8,9]. An alternative approach is recombinant vaccine technologies that rely on key viral structural proteins, such as HA, NA, nucleoprotein (NP), and membrane protein (M) [10-12]. Another approach is the use of DNA vaccines. Compared with other approaches, DNA vaccines are advantageous in that the development period for newly emerging influenza strain is shorter. Moreover, DNA vaccines are easier and cheaper to manufacture in a microbial host [13-15]. However, DNA vaccines have suffered from drawbacks, such as low gene-delivery efficiency and limited immunogenicity. 

In this study, we sought to improve the efficacy of DNA vaccines using a non-replicable baculovirus vector as a nano-carrier of antigen-encoding DNA. Recent accumulating evidence supports the notion that baculoviruses carrying mammalian cell-active promoters can express foreign genes in a variety of primary and established mammalian cells, as well as in animal models [16,17]. Thanks to their highly efficient gene-delivery mechanism, baculoviruses have stimulated increasing interest as a novel vector for vaccine development [18]. Here, we hypothesized that a non-replicable baculovirus vector carrying swine influenza H1N1 (sH1N1) HA would provide immune responses comparable to that of conventional, whole killed virus-based vaccines. To improve targeted HA gene delivery, we incorporated the envelope glycoprotein of human endogenous retrovirus (HERV-W) in our recombinant baculovirus [19,20]. The efficacy of the resulting baculoviral vector-based HA vaccine, AcHERV-sH1N1-HA, was tested in two strains of mice, BALB/C and C57BL/6, which are widely used as animal models for influenza virus research [21]. In addition to humoral and cellular immune responses, challenge tests were performed with pandemic H1N1 virus. 

## Materials and Methods

### Cells and virus

293TT cells were cultured in Dulbecco’s modified Eagle medium (DMEM) supplemented with 10% fetal bovine serum (Gibco BRL, USA) at 37°C in a humidified 5% CO_2_ incubator. Sf9 cells were maintained in Sf-900 medium (Invitrogen Co., USA) at 27°C. Influenza virus A/California/04/2009(H1N1) was used for immune response and challenge tests. 

### Animals

Female BALB/C and C57 BL/6 mice, purchased from Orient-Bio (Korea), were housed under filter-top conditions and provided water and food *ad libitum*. Mice were maintained in accordance with the Guide for the Care and Use of Laboratory Animals and housed in a Bio-safety Level 2 facility. The use of animals in these experiments was approved by the Institutional Animal Care and Use Committee (IACUC) of Konkuk University (Approval No.: KU12078). 

### Construction of recombinant baculovirus

**Figure 1:**
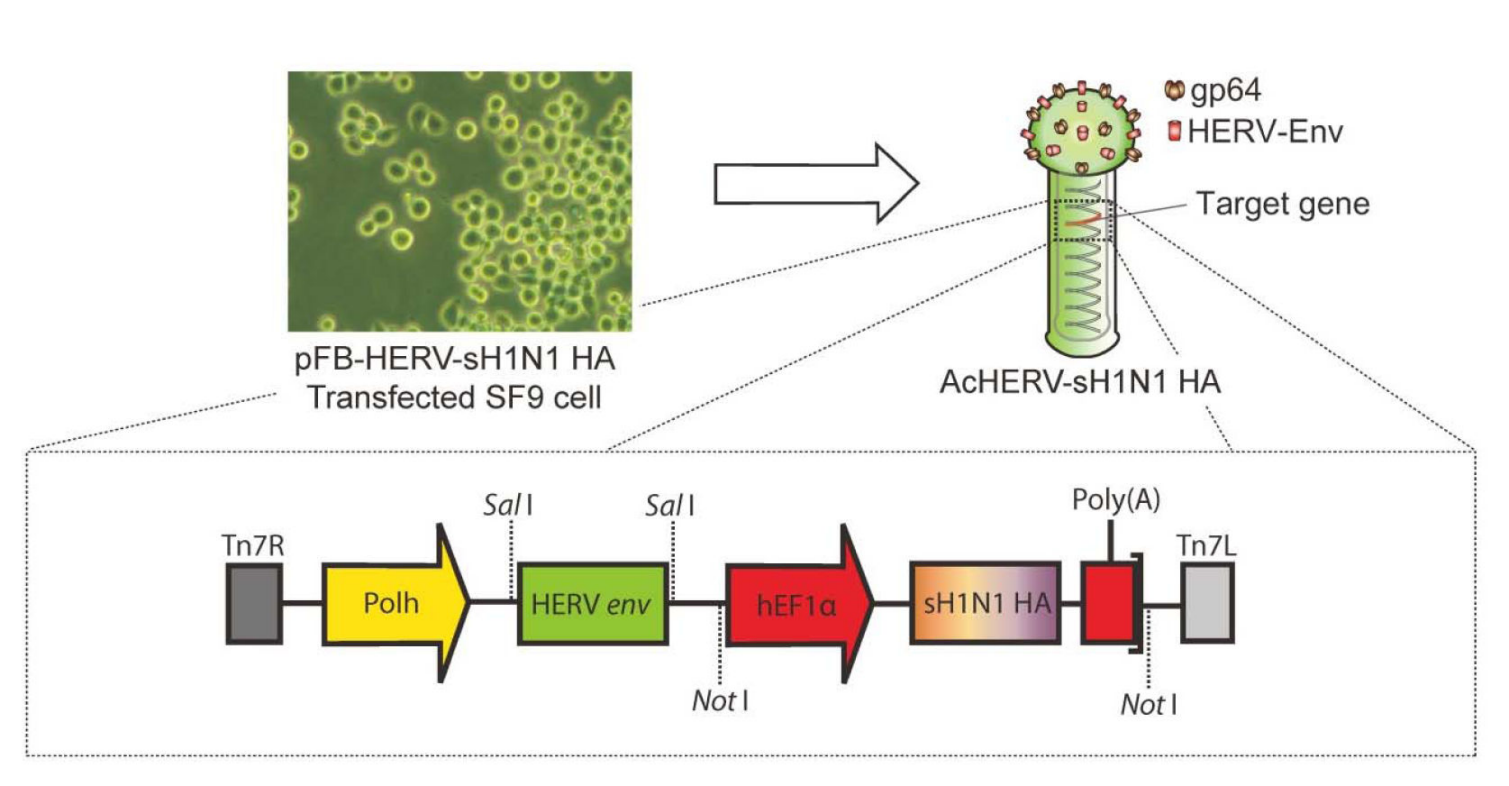
Schematic diagrams of recombinant baculoviruses expressing sH1N1 HA. A pFBHERV-sH1N1 vector containing HERV *env* and sH1N1 HA genes under the control of a polyhedron promoter and hEF1α promoter, respectively, was constructed. The recombinant baculovirus, AcHERV-sH1N1-HA, was generated using the Bac-to-Bac baculovirus expression system.

A recombinant baculovirus vector expressing HERV *env* (pFastBac-HERV) was previously constructed by inserting a synthetic, codon-optimized envelope gene of HERV type W (GenBank accession number NM014590; GenScript Corp., USA) into pFastBac (Invitrogen, USA)[19]. The baculovirus vaccine was constructed by synthesizing the full-length sH1N1 HA gene from A/California/04/2009(H1N1) (GenBank accession no. GQ117044.1) and introducing it into *Nco*I and *Nhe*I sites of pFastBac-HERV. Recombinant baculovirus was produced using the Bac-to-Bac baculovirus expression system (Invitrogen, USA), according to the manufacturer’s manual. The scheme for constructing the recombinant baculovirus, AcHERV-sH1N1-HA, is shown in Figure 1.

### Characterization of AcHERV-sH1N1-HA and its expression in mammalian cells

The construction of recombinant baculoviral vector vaccine was confirmed by isolating viral genomic DNA from recombinant baculovirus, propagated in Sf9 cells using a Nucleospin RNA virus kit (Macherey-Nagel, Germany). The HA and HERV genes were amplified by polymerase chain reaction (PCR). Primers for HA were 5’-taaaagccatggaaatgaaggcaatactag-3’ for sense and 5’- tgctagctttaaccgtaccatccatctac-3’ for antisense. Primers for HERV was 5’-tttgttcgcccaggactcta-3’ for sense and 5’-gtttaccccgcgccaccttctctaggca-3’ for antisense. The expression of AcHERV-sH1N1-HA in mammalian cells was tested by infecting 293TT cells with AcHERV-sH1N1-HA at a multiplicity of infection (MOI) of 10. Seventy-two hours after infection, 293TT cells were harvested and cell lysates were separated by sodium dodecyl sulfate-polyacrylamide gel electrophoresis (SDS-PAGE) on a 10% gel and transferred to a nitrocellulose membrane. The membrane was incubated with anti-sH1N1 sera (1:400 dilution) obtained from a mouse immunized with a commercially available killed sH1N1 vaccine (Greenflu; Green Cross Co., Korea), kindly provided by the manufacturer. An anti-ß-actin antibody (1:1,000 dilution; Santa Cruz Biotechnology, USA) was used as a protein loading control. Horseradish peroxidase (HRP)-conjugated monoclonal goat anti-mouse was used as a secondary antibody (1:2,000 dilution; Santa Cruz Biotechnology). 

For the detection of HA expression by immunofluorescence assay, confluent monolayers of 293TT cells grown on cover glasses in 4-well plates were infected with AcHERV-sH1N1-HA at an MOI of 10. Cells were stained by incubating with anti-HA primary antibody (1:200 dilution) and fluorescein isothiocyanate-conjugated goat anti-mouse secondary antibody (1:500 dilution; Santa Cruz Biotechnology). The cells were observed under a confocal laser-scanning microscope (LSM710, Carl Zeiss). 

### Immunization

Five-week-old BALB/C and C57BL/6 mice (15 mice per group) were immunized by intramuscular (I.M.) injection of 5 × 10^7^ plaque-forming units (PFU) of AcHERV-sH1N1-HA or 2 µg of the commercially available killed virus vaccine Greenflu at 0, 2, and 4 weeks. Mice in a third group (controls) were injected I.M. with phosphate-buffered saline (PBS) at the same time points. Serum samples were collected 1, 3, and 5 weeks after immunization to screen for humoral immune responses. Two weeks after the final booster injection, mouse splenocytes were isolated from two mice from each group for cellular immune response screening. Mice were intranasally challenged with 10-times the median lethal dose (MLD_50_) of influenza virus A/California/04/09 (H1N1) and monitored for clinical signs for 14 days. Three mice from each group were sacrificed 7 days after challenge, at which point their virus titers were analyzed and lung tissues were stained with hematoxylin-eosin (HE). 

### Hemagglutination inhibition (HAI) assay

Anti-HA antibody titers were measured by HAI assay. Sera from immunized mice were serially diluted (two fold) in V-shaped 96-well plates. Four hemagglutination units (HAU) of virus were added to the test and incubated at room temperature for 45 minutes. Thereafter, 1% chicken red blood cells were added and incubated at room temperature for 30 minutes. The HAI titer is the reciprocal of the highest serum dilution that completely inhibits hemagglutination [22].

### Enzyme-linked immunosorbent assay (ELISA)

An aliquot (60 μl) of inactivated, purified influenza virus A/California/04/2009(H1N1) (8 HAU/mL) was coated onto each well of a 96-well plate and incubated for 16 hours at 4°C. The plates were then washed and blocked with 2% (w/v) bovine serum albumin in PBS. Serially diluted mouse serum was added and incubated at room temperature for 2 hours. Following washing, HRP-conjugated goat anti-mouse IgG antibody (1:2000, Santa Cruz Biotechnology) was added and incubated for 1 hour at 37°C. TMB (3, 3’, 5, 5’-tetramethyl benzidine) substrate solution (Pierce, USA) was added, and color development was monitored at 450 nm. Results were expressed as reciprocals of the final detectable dilution. The titer values were expressed as a mean ± standard deviation (SD).

### Enzyme-linked immunospot (ELISPOT) assay

The production of interferon gamma (IFN-γ) from splenocytes of immunized mice was detected by ELISPOT assay. A 96-well plate was coated with 0.2 μg of anti-mouse IFN-γ capture antibody and then blocked with 10% FBS at 37°C. Splenocytes (2 × 10^6^ cells/well) were seeded in 100 μl of RPMI-1640 medium, stimulated with inactivated influenza virus A/California/04/2009(H1N1), and incubated for an additional 24 hours at 37°C. Plates were washed with PBS containing 0.05% Tween-20 and treated with 20 ng of biotinylated anti-mouse IFN-γ detection antibody. After 2 hours, streptavidin-alkaline phosphatase was added to the wells, and color was developed with an AEC substrate reagent (BD Biosciences, USA). The number of spots was counted using an ELISPOT reader (AID ElispotReader ver.4, Germany). The ELISPOT numbers were expressed as a mean ± standard deviation (SD).

### Challenge

After 6 weeks, immunized mice were challenged intranasally with 10 MLD_50_ (based on the lethal dose in C57BL/6) influenza virus A/California/04/2009(H1N1). Body weight was monitored every day for 14 days. Mice were sacrificed for analyses of virus titer in the lung and HE staining was carried out. After final monitoring, all the survived mice were humanely euthanized using cervical dislocation according to the AVMA guidelines for the euthanasia of animals. 

### Statistical analysis

Statistical analyses were performed using SigmaPlot 11.0 software (Systat Software, USA). Where indicated, statistical significance was assessed using Student’s *t*-test. P-values ≤ 0.05 were considered significant.

## Results

### Expression of HA protein from AcHERV-sH1N1-HA recombinant baculovirus *in vitro*

**Figure 2:**
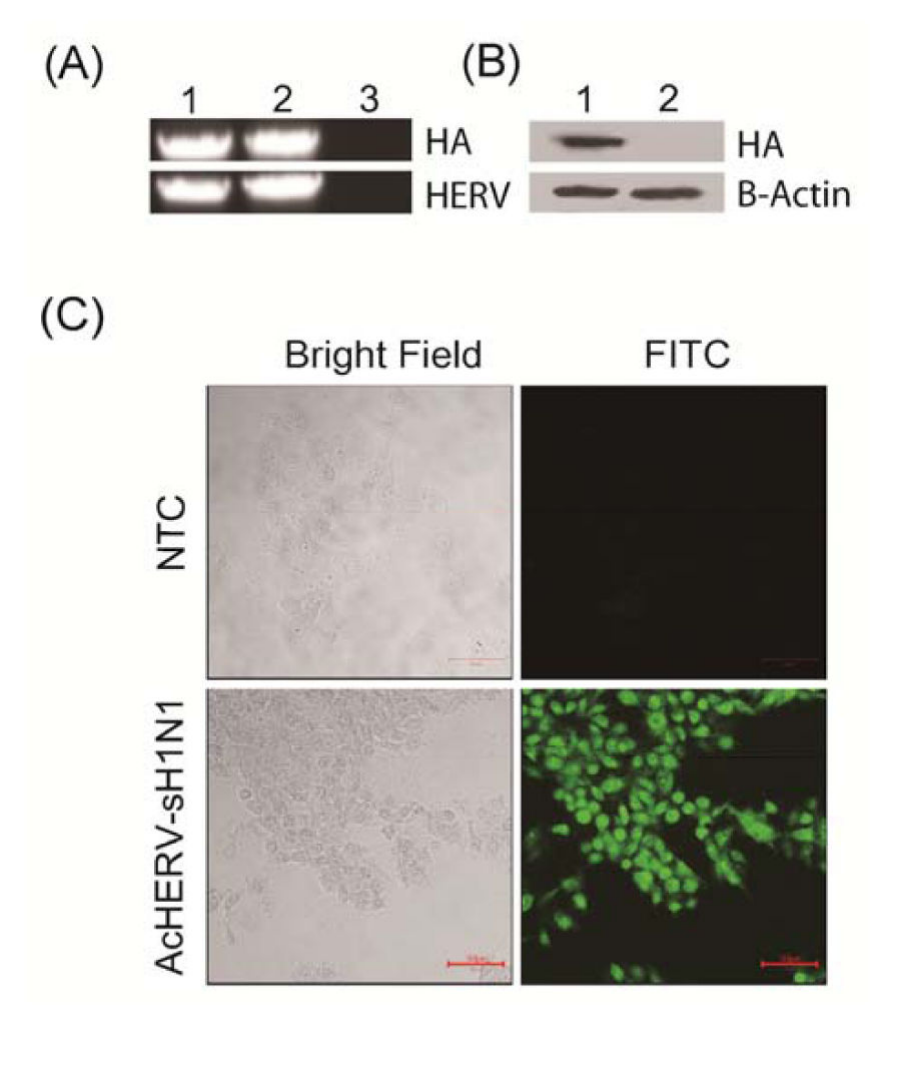
Characterization of the recombinant baculovirus, AcHERV-sH1N1-HA, and its expression in mammalian cells. (A) PCR detection of HA and HERV. (1) pFastBacHERV-sH1N1-HA; (2) viral DNA for AcHERV-sH1N1-HA; (3) negative control. (B) Western blot analysis of HA protein expression in 293TT cells. (1) Lysates of AcHERV-sH1N1-HA–infected 293TT cells; (2) lysates of mock-infected cells. (C) Immunofluorescence detection of HA protein in 293TT cells.

AcHERV-sH1N1-HA construction and its expression in mammalian cells were tested. The construction of recombinant baculovirus AcHERV-sH1N1-HA was confirmed by amplification of HERV and HA genes by PCR, which showed that the HERV envelope gene and sH1N1 HA gene were correctly inserted in the AcHERV-sH1N1-HA construct (Figure 2A). Western blotting of AcHERV-sH1N1-HA–infected 293TT cells revealed the expression of sH1N1 HA protein (~63 kDa) 48 hours post infection (Figure 2B). The intracellular expression of sH1N1 HA protein in mammalian 293TT cells was confirmed by immunofluorescence analysis (Figure 2C). 

### Cellular immune responses in mice

**Figure 3:**
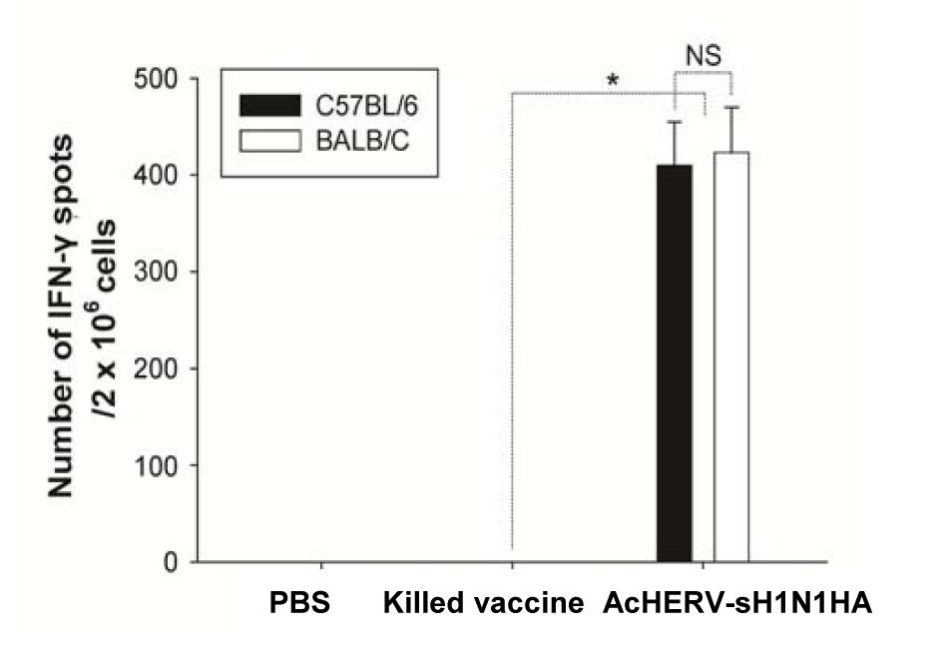
Cellular immune responses in mice immunized with AcHERV-sH1N1-HA or killed virus vaccine. Mouse splenocytes were harvested 2 weeks after the final immunization. The number of IFN-γ–producing sH1N1 HA-specific T cells was determined using ELISPOT assays. *P<0.05 versus killed virus vaccine group; not significant (NS) versus the number of IFN-γ spots in the BALB/C group.

The AcHERV-sH1N1-HA vaccine, but not the killed virus vaccine, induced distinct levels of cellular immune responses in mice after intramuscular immunization. In mice immunized with the killed virus vaccine, there was no detectable IFN-γ production by splenocytes stimulated with pandemic influenza H1N1 virion. Unlike mice immunized with killed virus vaccines, mice treated with the AcHERV-sH1N1-HA vaccine showed 410 ± 45 and 423±47 IFN-γ spots per 2 × 10^6^ splenocytes for C57BL/6 and BALB/C mice, respectively (Figure 3). There was no significant difference in IFN-γ production levels between BALB/C and C57BL/6 mice immunized with killed virus or AcHERV-sH1N1-HA vaccine.

### Humoral immune responses in mice

**Figure 4:**
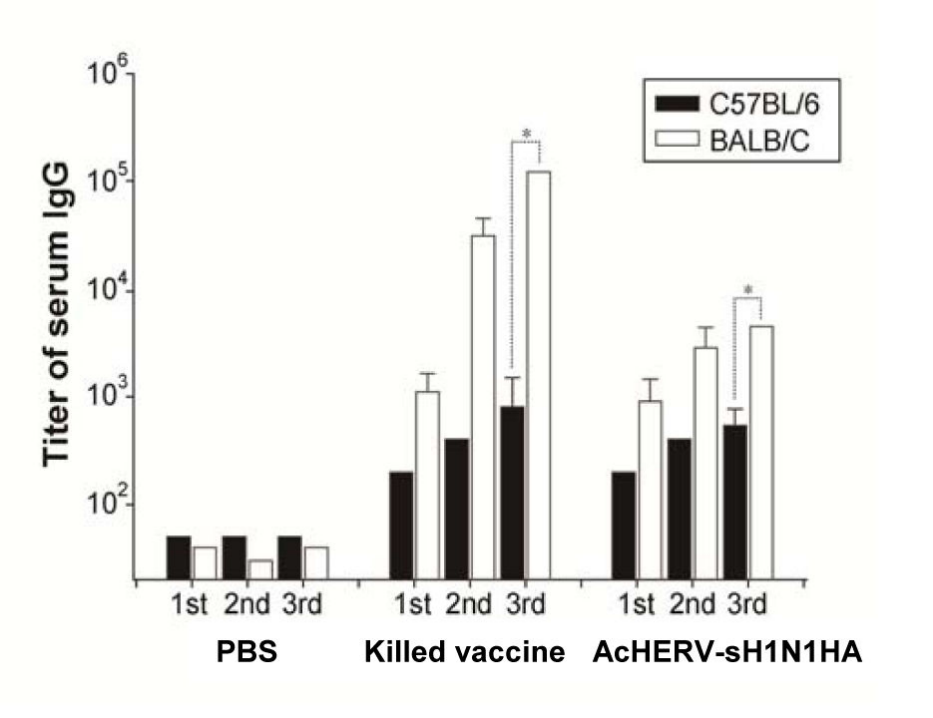
Humoral immune response of mice immunized with AcHERV-sH1N1-HA or killed virus vaccine. BALB/C and C57BL/6 mice were intramuscularly immunized with 5 × 10^7^ PFU of recombinant AcHERV-sH1N1-HA, killed virus vaccine, or PBS. Antigen-specific IgG antibody titers against HA in BALB/C and C57BL/6 were determined by ELISA *P<0.05, versus BALB/C third immunization titer.

**Figure 5:**
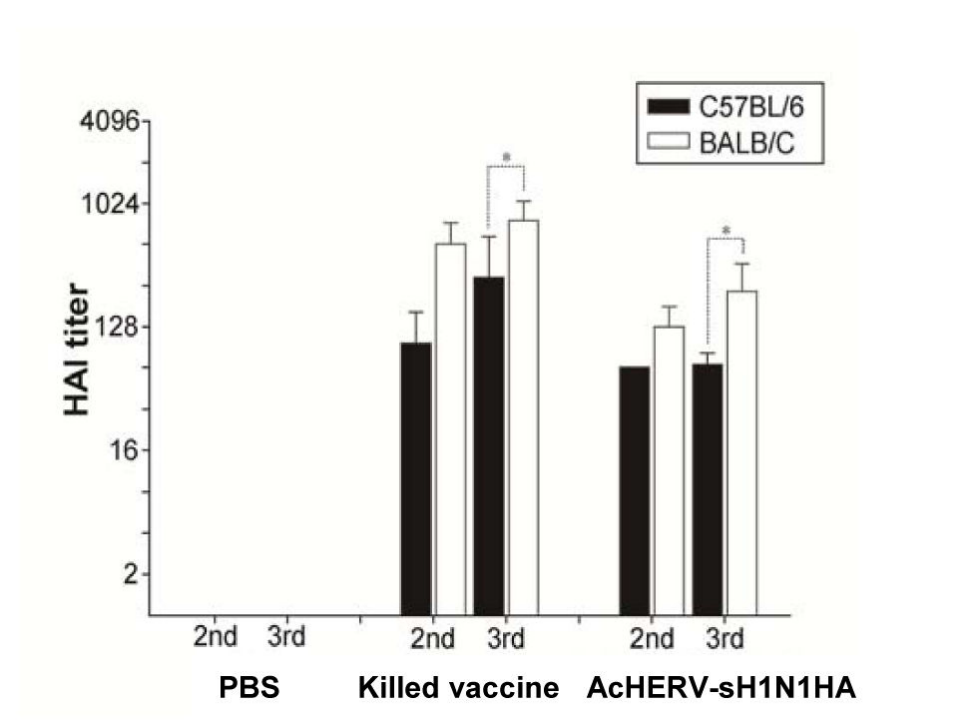
HAI assays with A/California/04/2009(H1N1). Four HAUs of A/California/04/2009(H1N1) virus was seeded into a V-shape, 96-well plate and combined with 2-fold serially diluted serum. After antigen-antibody reaction, 1% chicken blood was added for determination of HAI titer. *P<0.05, versus BALB/C third HAI titer.

Unlike cellular immune responses, humoral immune responses were mouse strain dependent. Regardless of vaccine type, BALB/C mice showed significantly higher levels of HA-specific serum IgG levels than C57BL/6 mice. In the case of killed virus vaccine-immunized groups, the serum IgG titers of BALB/C mice were more than two orders of magnitude higher than those of C57BL/6 mice after the third booster (Figure 4). The serum IgG titers of AcHERV-sH1N1-HA–immunized BALB/C mice were ten-fold higher than those of C57BL/6 mice after the final boost. Consistent with the serum IgG titers, HAI assays showed higher inhibition capacity of BALB/C mice relative to C57BL mice following immunization with killed virus or AcHERV-sH1N1-HA vaccine (Figure 5). The exact titer values following killed virus immunization were 768.0 ± 295.6 for BALB/C, and 294.0 ±298.1 for C57BL/6. Similarly, the titer values after AcHERV-sH1N1-HA vaccine immunization were 233.6±139.7 for BALB/C, and 67.1±14.3 for C57BL/6.

### Protection against challenge in mice

**Figure 6:**
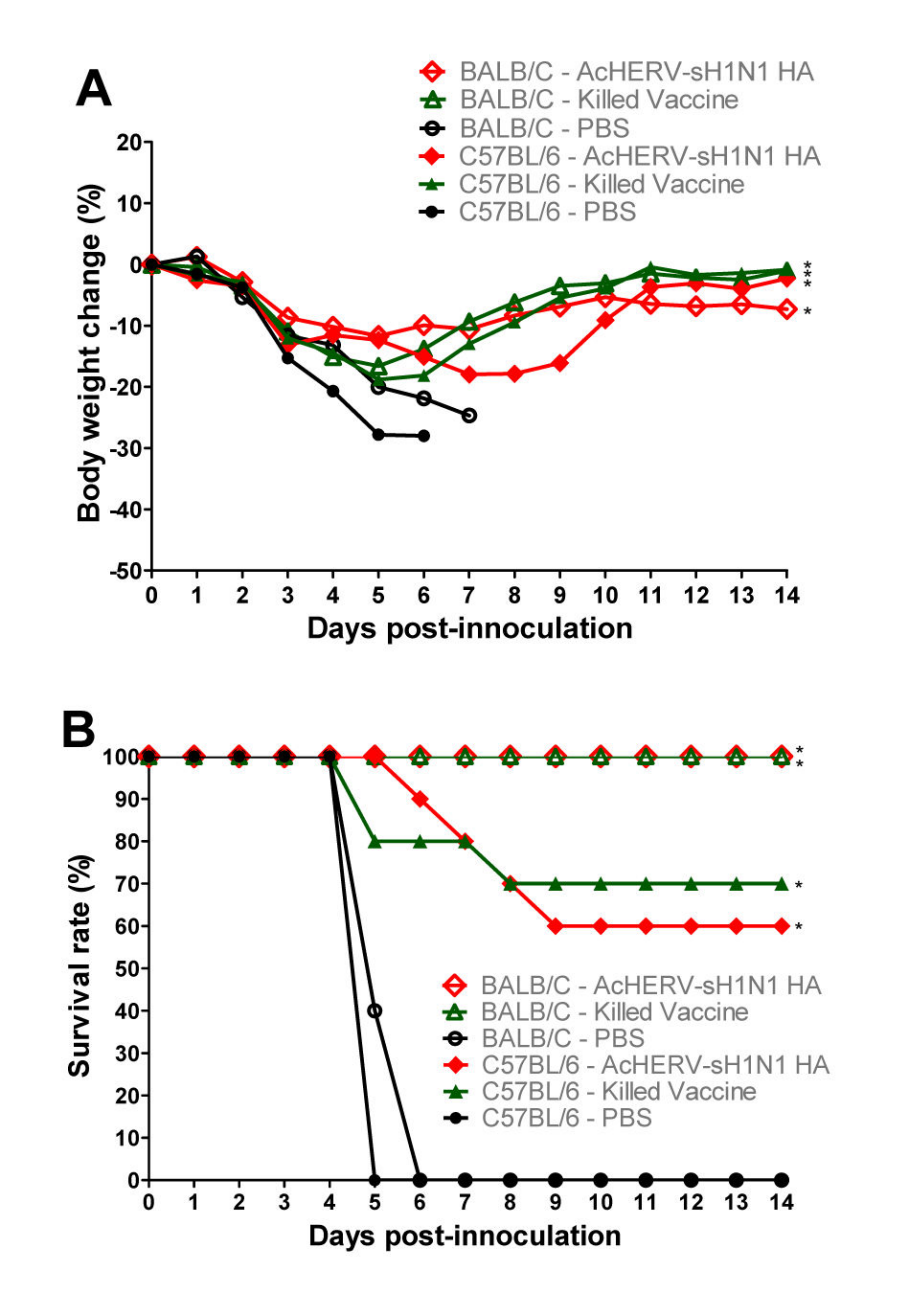
Protective efficacy of AcHERV-sH1N1-HA vaccine against lethal influenza challenge. (A) Percentage change in body weight after challenge in mice. Changes in body weight (n=10) are expressed as the mean in each group. (B) Survival rate after challenge in mice. Symbols: AcHERV-sH1N1-HA–vaccinated C57BL/6, filled red diamonds; AcHERV-sH1N1-HA–vaccinated BALB/C, open red diamonds; killed virus-vaccinated C57BL/6, filled green triangles; killed virus-vaccinated BALB/C, open green triangles; PBS-injected C57BL/6, filled grey circles; PBS-injected BALB/C, open grey circles. *P<0.05 compared with PBS-injected mice; repeated measures ANOVA from day 1 to day 14 post infection.

In mice, the AcHERV-sH1N1-HA vaccine provided protective immunity against influenza virus A/California/04/2009(H1N1) comparable to that of the killed virus vaccine. Similar to the humoral immune responses, murine strain dependence was observed for the degree of protection after challenge. In C57BL/6 mice immunized with the killed virus vaccine, the survival rate was 70% at 14 days post-challenge, whereas that of AcHERV-sH1N1-HA vaccine-immunized mice was 60% (Figure 6B). Unlike C57BL/6 mice, BALB/C mice showed 100% survival rates after challenge. Immunization of BALB/C mice with either killed virus vaccine or AcHERV-sH1N1-HA vaccine was effective in providing complete protection against influenza virus A/California/04/2009(H1N1). 

The body weights of mice immunized with the killed virus vaccine or AcHERV-sH1N1-HA vaccine decreased gradually after challenge, but recovered beginning at 6 days. Mice treated with PBS showed a continuous reduction in body weight after challenge, and all had reached the death point (over 20% weight loss by 6 days after challenge (Figure 6A). 

**Figure 7:**
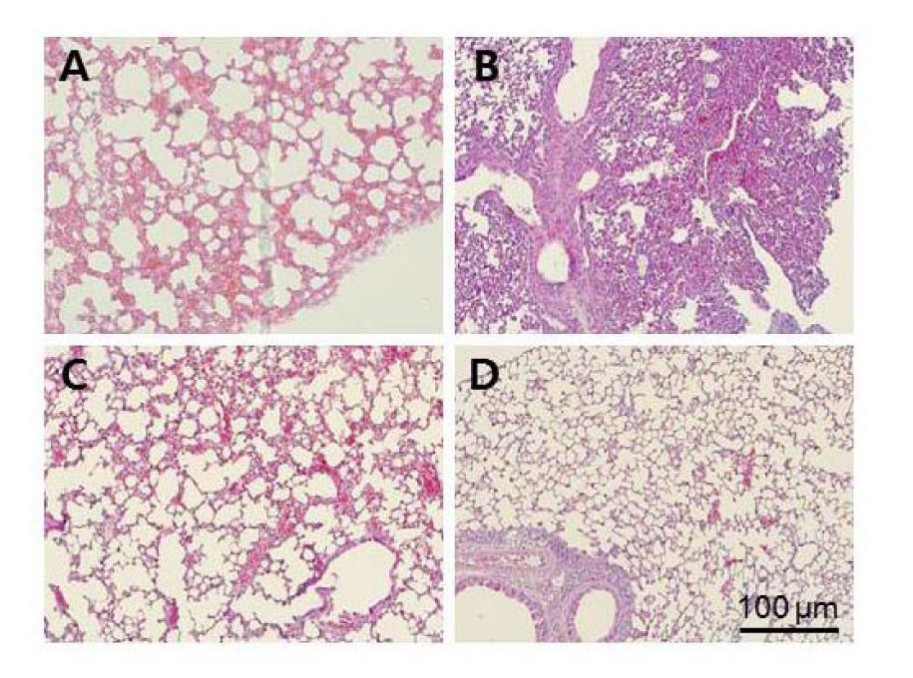
Histological lesions in the lung sections of immunized C57BL/6 mice after challenge with A/California/04/2009(H1N1). Seven days post challenge, mice in each group were euthanized and their lungs were HE-stained for histological evaluation. (A) Normal C57BL/6 mice. (B) Non-vaccinated C57BL/6 mice challenged with virus. (C) C57BL/6 mice vaccinated with commercial killed virus vaccine. (D) C57BL/6 mice vaccinated with AcHERV-sH1N1-HA.

To investigate virus shedding in challenged mice, virus titers in lung fluid were determined in eggs and expressed as log_10_ EID_50_/ml. As shown in Table 1, vaccine-immunized BALB/C mice showed no virus shedding. Consistent with the survival data, non-immunized mice shed virus, exhibiting mean titers of 5.4 ± 0.0 log_10_ EDI50/ml. However, the virus titers in the lungs of C57BL/6 mice were not changed with or without immunization. Consistent with the survival data, histological analyses revealed that lung tissues were damaged in PBS-treated C57BL/6 mice (Figure 7B). However, the lung tissue of mice immunized with the killed virus vaccine (Figure 7C) or AcHERV-sH1N1-HA vaccine (Figure 7D) showed less damage compared to that of non-immunized mice (Figure 7A).

**Table 1:** Lung titers of challenge virus in mice at 7 days post challenge.

**Immunized Group**	**Lung virus titers ^a^**	
	**BALB/C**	**C57BL/6**
PBS	5.5 ± 0.0 (3/3)	5.5 ± 0.0 (3/3)
Killed Vaccine	< ^b^ (0/3)	5.4 ± 0.0 (3/3)
AcHERV–sH1N1 HA	< ^b^ (0/3)	5.4 ± 0.0 (3/3)

^a^ Virus titers were determined in eggs and are expressed as log_10_ EID_50_/ml. Data are presented as means ± SD of titers of positive samples (≥0.75 log_10_ EID_50_/ml). The numbers of mice that shed virus are indicated in parentheses (number shedding/number tested). ^b^ Virus was not detected in the lungs**.**

## Discussion

In this study, we demonstrated the importance of humoral immune responses as a major defense system against influenza viral challenge. The complete survival of BALB/C mice immunized with AcHERV-sH1N1-HA after challenge with sH1N1 virus suggests the feasibility of HERV envelope-coated, recombinant baculoviral vector-based HA vaccines as an alternative to killed virus vaccines against seasonal influenza pandemics. 

As an H1N1 antigen gene-delivery vector, we used the baculovirus *Autographa californica* multiple nuclear polyhedrosis virus expression system. Baculovirus has been studied as an effective delivery vehicle for transgene expression in mammalian cells, including human cells [17,23,24]. Recombinant baculoviruses containing mammalian cell promoters can provide high-level foreign gene expression in mammalian cells [25-27]. However, insect cell-specific baculoviruses have suffered from limited delivery into mammalian cells [28]. 

 In a previous study, pantropic VSV-G envelope protein was embedded on the surface of baculovirus to enhance the infection of mammalian cells by baculoviral vectors [15]. The resulting recombinant baculoviral vector vaccine expressing both VSV-G envelope and influenza HA evoked both humoral and cellular immune responses and provided effective protection against lethal virus challenge in BALB/C mice and chicken hosts [18]. However, the high cytotoxicity of VSV-G protein [29] and its immediate inactivation by serum complement systems hinders the application of this system as a DNA vaccine delivery vector [28]. In contrast to this previous approach, we introduced a HERV envelope-encoding gene into the baculoviral genome to improve baculovirus delivery into human cells, as shown in Figure 1. We recently reported that a human papillomavirus L1 gene-encoding, HERV envelope-coated baculoviral vector vaccine significantly improved the expression of human papillomavirus L1 protein in mammalian cells and induced humoral immune responses comparable to those of commercially available cervical cancer virus-like particle vaccines [16,17].

Notably, we observed that the humoral, but not cellular, immune responses of the AcHERV-sH1N1-HA vaccine depended on the strain of mice. C57BL/6 and BALB/C mice have been widely used as animal models to evaluate the efficacy of various experimental vaccines. Because of the strong Th1 and Th2 immune responses, BALB/C mice have been generally used for evaluation of vaccine efficacy. Unlike BALB/C, C57BL/6 has been used for Th1 based immune study such as cancer therapy [30]. The difference of immune response and susceptibility between BALB/C and C57BL/6 were found to be related to the differences in cytokine profiles and dendrite cell subsets [31]. In addition, there exists possibility that the strain dependence would be in part attributed by the nature of transgene. For antigens such as lipopolysaccharide, lipoprotein and CpG, the production of interleukin-12 p40 was found to be higher in the dendritic cells of C57BL/6 as compared to those of BALB/C [32]. For stress-inducible 1 protein antigen of *Leishmania major*, higher induction of Th1 CD4+ T cell immune response was observed in BALB/C as compared to C57BL/6 mice [33]

We observed that the AcHERV-sH1N1-HA vaccine induced substantially greater cellular immune responses (Figure 4) compared to that of the killed virus vaccine, but provided a comparable degree of protection (Figure 6). The lack of correlation between cellular immune response and degree of protection suggests the importance of the humoral immune response as the main defense mechanism against influenza virus infection. Previously, adenoviral vector-based influenza HA vaccines were reported to induce both cellular and humoral immune responses. In this study, the cellular immune response was suggested to play an important role in conferring protection against different subtypes of influenza virus infection. However, the study in question used a single strain of BALB/C mice to evaluate cellular and humoral immune responses, and did not compare results with those obtained using killed virus vaccines, which do not evoke cellular immune responses [29]. 

Although we did not evaluate the long-term immune responses after AcHERV-sH1N1-HA vaccination, it is likely that AcHERV-sH1N1-HA vaccine may provide the long-term immune responses. In our previous study, the humoral immune responses to human papillomavirus 16 L1 antigen were sustained at least up to 20 months in BALB/C mice after vaccination with AcHERV encoding human papillomavirus 16 L1 antigen [19]. The comparison of long-term immune response between AcHERV-sH1N1-HA and the seasonal vaccine needs to be done further. 

We provided evidence that the AcHERV-sH1N1-HA vaccine offers an immunogenicity and protective efficacy comparable to that of killed virus vaccines. As a baculoviral vector-based vaccine, the AcHERV-sH1N1-HA vaccine has advantages in terms of ease of manipulation, simple scale-up, and lack of toxicity. However, there are challenges to further improving the AcHERV-sH1N1-HA vaccine. Unlike killed virus vaccines, which contain all the components of the influenza virus, AcHERV-sH1N1-HA only elicits an immune response to HA proteins. Such monovalency may limit the degree of protection against specific type A influenza viruses. Polyvalent AcHERV-sH1N1 vaccines encoding HA, neuraminidase, and M proteins that may overcome this limitation are currently under construction. 

As compared to a naked DNA vaccine, the AcHERV-sH1N1-HA vaccine has several advantages. First, the entrapment of antigen gene in the recombinant baculoviral vector can improve the in vivo stability and significantly alter pharmacokinetic behavior. In our previous study, we reported that the mean residence times of human papillomavirus 16 L1 gene delivered in AcHERV baculoviral vector were 4.8 and 272.2-fold higher than naked human papillomavirus 16 L1 DNA vaccines [34]. Secondly, the existence of HERV moiety on the surface of the baculoviral vector can enhance the cellular uptake and antigen expression in the cells. In general, the intracellular delivery of naked plasmid DNA has suffered from the lack of cellular uptake due to the large molecular size and high negative charges of plasmid DNA. The intracellular antigen protein expression levels of gene delivered by AcHERV vector was shown to be higher than those delivered by the wild baculoviral vector [19]. 

In conclusion, a baculovirus-based AcHERV-sH1N1-HA vaccine induced cellular and humoral immune responses in mice. The protective efficacy of the AcHERV-sH1N1-HA vaccine against influenza A/California/04/2009(H1N1) was comparable to that of a commercial killed virus vaccine, suggesting the potential of HERV envelope-coated, recombinant baculovirus-based HA vaccines against pandemic influenza H1N1 virus.
